# Assessing children who are acutely ill in general practice using the National PEWS and LqSOFA clinical scores: a retrospective cohort study

**DOI:** 10.3399/BJGP.2023.0638

**Published:** 2024-10-15

**Authors:** Amy Clark, Rebecca Cannings-John, Enitan D Carrol, Emma Thomas-Jones, Gerri Sefton, Alastair D Hay, Christopher C Butler, Kathryn Hughes

**Affiliations:** Paediatrics, Cambridge University Hospitals NHS Foundation Trust, Cambridge.; Centre for Trials Research, Cardiff University, Cardiff.; Department of Clinical Infection, Microbiology and Immunology, Institute of Infection, Veterinary and Ecological Sciences, University of Liverpool, Liverpool; Infectious Diseases Department, Alder Hey Children’s NHS Foundation Trust, Liverpool.; Centre for Trials Research, Cardiff University, Cardiff.; Alder Hey Children’s NHS Foundation Trust, Liverpool.; Population Health Sciences, Bristol Medical School, University of Bristol, Bristol.; Nuffield Department of Primary Care Health Sciences, University of Oxford, Oxford.; PRIME Centre Wales, Division of Population Medicine, Cardiff University, Cardiff.

**Keywords:** child health, clinical prediction rule, early warning score, general practice, retrospective studies, triage

## Abstract

**Background:**

Clinical tools are needed in general practice to help identify children who are seriously ill. The Liverpool quick Sequential Organ Failure Assessment (LqSOFA) was validated in an emergency department and performed well. The National Paediatric Early Warning System (PEWS) has been introduced in hospitals throughout England with hopes for implementation in general practice.

**Aim:**

To validate the LqSOFA and National PEWS in general practice.

**Design and setting:**

Secondary analysis of 6703 children aged <5 years presenting to 225 general practices in England and Wales with acute illnesses, linked to hospital data.

**Method:**

Variables from the LqSOFA and National PEWS were mapped onto study data to calculate score totals. A primary outcome of admission within 2 days of GP consultation was used to calculate sensitivity, specificity, negative predictive values (NPVs), positive predictive values (PPVs), and area under the receiver operating characteristic curve (AUC).

**Results:**

A total of 104/6703 children were admitted to hospital within 2 days (pre-test probability 1.6%) of GP consultation. The sensitivity of the LqSOFA was 30.6% (95% confidence interval [CI] = 21.8% to 41.0%), with a specificity of 84.7% (95% CI = 83.7% to 85.6%), PPV of 3.0% (95% CI = 2.1% to 4.4%), NPV of 98.7% (95% CI = 98.4% to 99.0%), and AUC of 0.58 (95% CI = 0.53 to 0.63). The sensitivity of the National PEWS was 81.0% (95% CI = 71.0% to 88.1%), with a specificity of 32.5% (95% CI = 31.2% to 33.8%), PPV of 1.9% (95% CI = 1.5% to 2.5%), NPV of 99.1% (95% CI = 98.4% to 99.4%), and AUC of 0.66 (95% CI = 0.59 to 0.72).

**Conclusion:**

Although the NPVs appear useful, owing to low pre-test probabilities rather than discriminative ability, neither tool accurately identified admissions to hospital. Unconsidered use by GPs could result in unsustainable referrals.

## Introduction

Life-threatening illnesses in children, such as meningitis and meningococcal sepsis, are declining in incidence; however, emergency hospital admissions in this patient group are increasing annually.^1^^–^^6^ The assessment of children who are acutely unwell can be challenging in general practice because of vague systemic symptoms and the low prevalence of serious illness.^6^ It can be difficult for GPs to identify children who can be safely managed at home while also identifying the few who are at risk of serious illness and need hospital admission.

Various clinical scoring systems have been developed to help clinicians. The National Institute for Health and Care Excellence (NICE) ‘traffic light’ system was recommended for use in general practice but, until recently, had not been validated in this setting. It has now been found to perform poorly on account of categorising almost all children as being at ‘moderate’ or ‘high’ risk of serious illness and displaying low sensitivity and specificity.^7^ Clinical prediction tools developed in hospital settings may also perform well in general practice but it is essential that these are tested and validated in general practice before implementation.

One promising tool developed in a paediatric emergency department is the Liverpool quick Sequential Organ Failure Assessment (LqSOFA) score, developed to identify life-threatening infections in febrile children.^8^ This tool has a good prognostic ability for detecting critical care admissions, consisting of four variables: heart rate, respiratory rate, consciousness level, and capillary refill time.

**Table:** How this fits in

The National Institute for Health and Care Excellence-recommended traffic light system for identifying seriously ill children has been found to perform poorly in general practice. A new National Paediatric Early Warning System (PEWS) has recently been introduced in hospitals. This study examined the performance of the National PEWS in general practice and found that it could not accurately identify children requiring hospital admission within 2 days of presenting to general practice with an acute illness and therefore should not be recommended for use in general practice without adjustment. Another score, the Liverpool quick Sequential Organ Failure Assessment (Lq-SOFA), was also found to perform poorly in general practice.

Most hospitals throughout the UK have a Paediatric Early Warning System (PEWS) to identify children at risk of deterioration. A standardised National PEWS has been developed by the Royal College of Paediatrics and Child Health, the Royal College of Nursing, and NHS England.^9^ This score has recently been introduced in hospitals throughout England and it is hoped that eventually a modified version might be suitable for implementation in general practice.^9^ If the new National PEWS could be adopted in general practice it could bridge the gap in continuity between primary and secondary care, allowing a synergistic approach to the assessment of unwell children in a variety of settings.

Any clinical decision tools incorporated into general practice must be validated in this setting to ensure that clinicians understand the accuracy and utility of these scoring systems before using them to guide decisions. Such tools should have a high sensitivity, ensuring that all children with a serious illness are correctly ‘flagged’ and referred for secondary care assessment while providing reassurance that those who are not flagged can be safely managed at home. The aim of this study was to validate the LqSOFA and National PEWS within general practice.

## Method

This was a retrospective cohort study linking general practice study data with hospital admission data in England and Wales.

### Study participants

A secondary analysis of data from a previous study, the Diagnosis of Urinary Tract Infection in Young Children (DUTY) study, was performed.^10^ The DUTY study was a prospective cohort study analysing the presenting signs and symptoms of children aged <5 years in primary care who were acutely ill, to explore the features of urinary tract infections. The details of the DUTY study and the cohort demographics are reported elsewhere.^7^^,^^11^ Only participants presenting to general practice were included in the current study. The general practice study data were linked to routinely collected hospital data in England and Wales to identify admissions; provided by Hospital Episode Statistics (NHS Digital) and the Patient Episode Database for Wales (Secure Anonymised Information Linkage [SAIL] Databank).^7^^,^^11^^,^^12^

### Scores undergoing validation

The LqSOFA tool consists of four variables, each scoring one point if abnormal: heart rate, respiratory rate, consciousness level, and capillary refill time (CRT) (Supplementary Table S1).^8^ The National PEWS consists of four age-specific charts, with a maximum score total of 18: the charts for children aged 0–11 months (chart 1) and 1–5 years (chart 2) were used in this study (Supplementary Tables S2 and S3).

### Score calculations

Each criterion was matched to the variables available within the current study’s general practice dataset. Children with missing data for ≥2 of the scoring variables were excluded. If only one component was missing the child was included and the variable was assumed to be normal. This approach was used in the original LqSOFA and previous National PEWS articles.^8^^,^^13^

#### LqSOFA score matching

The matching of variables between the LqSOFA score and the current study’s dataset were discussed within the study team comprising of clinicians and senior researchers (Supplementary Table S1). The variables ‘heart rate’ and ‘respiratory rate’ were directly mapped onto the current study’s data, and the matching of consciousness level was unanimously agreed on by the authors. In the current study, ‘capillary refill time’ variable of 2–5 s could not be easily matched to the LqSOFA categories of <3 s (normal) or ≥3 s (abnormal). The authors consulted seven GPs and seven secondary care clinicians; all of the GPs supported classifying 2–5 s as abnormal, with four out of seven of the secondary care responders in agreement. Therefore, the authors classified CRT 2–5 s as abnormal.

#### National PEWS score matching

The matching of variables was agreed by the study team as described in Supplementary Table S4. The authors continued to classify CRT 2–5 s as abnormal. The authors excluded the PEWS variables ‘blood pressure’ and ‘oxygen requirement’ as these were not available in the current study’s general practice data.

### Outcome measures

#### Primary outcome

The primary outcome was a hospital admission within 2 days of the general practice index consultation, during which the child was recruited for the DUTY study. A ‘hospital admission’ was defined as a spell in hospital as an inpatient under the care of a consultant; assessment in the emergency department was not coded as an admission unless the treating team decided to admit them.

#### Secondary outcome

The secondary outcome was a composite outcome ‘serious illness episode’: either a serious illness diagnosed in hospital within 2 days of GP consultation or a hospital admission lasting ≥1 night within 2 days of GP consultation. The definition of ‘serious illness’ has been described previously and was based on the NICE definition within their fever guidelines.^7^^,^^14^

### Statistical analysis

The cohort was analysed descriptively to define the sample characteristics. This included general demographics (age, sex, number of days unwell, presence of fever, and score totals), hospital admissions, and comparison of children admitted and not admitted to hospital.

The test performance of the LqSOFA and National PEWS was then assessed. For each child, a score total was calculated using each assessment system, by adding up points for the constituent variables. PEWS totals were calculated separately using the age-specific charts of <12 months and 1–5 years. The age groups were then combined for analysis.

Area under the receiver operating characteristic curves (AUCs) were calculated for each of the scores. Sensitivity, specificity, positive predictive values (PPVs), negative predictive values (NPVs), and positive and negative likelihood values were calculated for each system, at each score threshold, alongside a 95% confidence interval (CI). Analyses were performed using IBM SPSS Statistics (version 25) and Stata (version 16.0). A sensitivity analysis was performed, restricting the cohort to febrile children, identified using the definition ‘measured or perceived elevation of body temperature above the normal daily variation (≥38°C) by a parent or clinician’. This was chosen to reflect the cohort characteristics of the original LqSOFA validation study.

## Results

There were 7163 children included in the original DUTY study. Those not recruited from general practices were excluded (*n* = 366), along with those where it was not possible to link them with their hospital data (*n* = 88) and without any clinical variables recorded (*n* = 6). This left a total of 6703 children from 225 general practices included in the study, whose demographics have been described previously.^7^

### LqSOFA

Data for ≥2 variables were missing in 1135/6703 (16.9%) children and they were excluded from the analysis (Supplementary Table S5). The most common missing variables were heart rate (22.7%, *n* = 1524) and respiratory rate (20.8%, *n* = 1391). Children excluded from the analysis were younger and less likely to be febrile, although no significant difference in hospital admissions was seen (Supplementary Table S6). Of the 6703 children, 5568 (83.1%) had either complete variables (*n* = 4508) or one variable missing (*n* = 1060), enabling an LqSOFA score to be calculated. The median age of included children was 2.2 years (Table 1).

**Table 1.** Demographics for included children who were scored on the LqSOFA, PEWS chart 1 (children aged <12 months), and PEWS chart 2 (children aged 12–60 months)

**Scoring system**	**Age, years, median (IQR)**	**Sex, *n* (%)**		**Days unwell, median (IQR)**	**Febrile children, *n* (%)**	**Primary outcome, *n* (%)**		**Secondary outcome, *n* (%)**	
	
		**Male**	**Female**			**All children**	**Febrile**	**All children**	**Febrile**
**LqSOFA (*n* = 5568)**	2.2 (1.0–3.5)	2704 (48.6)	2864 (51.4)	4 (3.0–7.0)	4221 (75.8)	85 (1.5)	79 (1.9)	42 (0.8)	42 (1.0)
**PEWS chart 1 (*n* = 1129)**	0.6 (0.4–0.8)	596 (52.8)	533 (47.2)	4 (3.0–7.0)	767 (67.9)	21 (1.9)	19 (2.5)	9 (0.8)	9 (1.2)
**PEWS chart 2 (*n* = 3770)**	2.9 (1.9–3.8)	1796 (47.6)	1974 (52.4)	4 (3.0–7.0)	2982 (79.1)	58 (1.5)	55 (1.8)	29 (0.8)	29 (1.0)

*IQR = interquartile range. LqSOFA = Liverpool quick Sequential Organ Failure Assessment. PEWS = Paediatric Early Warning System.*

**Figure 1.**
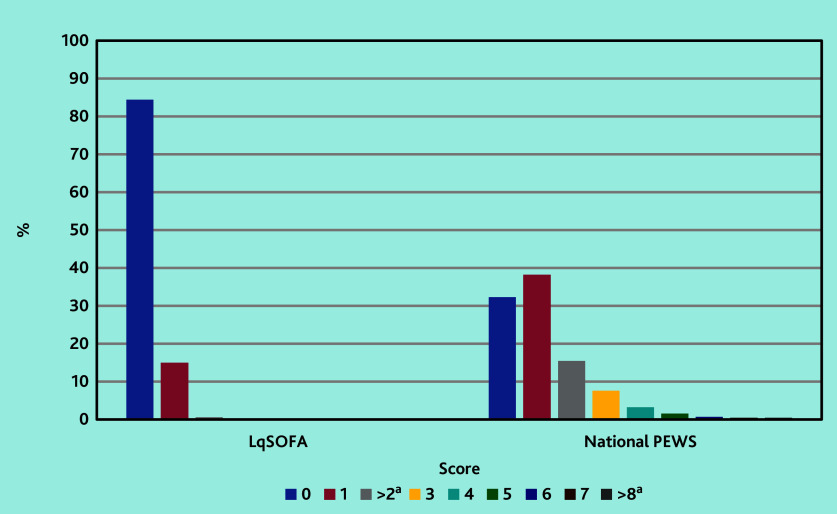
Graph demonstrating score frequencies for the LqSOFA and National PEWS. The majority of children are scoring either zero or one point. LqSOFA = Liverpool quick Sequential Organ Failure Assessment. National PEWS = National Paediatric Early Warning System. ^a^ For the LqSOFA, the scores >2 have been combined to adhere with the ‘small data’ reporting requirements of the Secure Anonymised Information Linkage (SAIL) Databank. For the National PEWS, the scores >8 have been combined.

The majority (84.4%, *n* = 4701/5568) of children scored 0 on the LqSOFA (Figure 1). The most common reason for scoring was a prolonged CRT, in 783/5568 (14.1%) children, followed by reduced conscious level in 55 (1.0%) children (data not shown).

### Primary outcome using LqSOFA

Of 5568 children, 85 (1.5%) were admitted to hospital within 2 days, and the AUC for predicting hospital admission was 0.58 (95% CI = 0.53 to 0.63, Table 2 and Supplementary Table S7). Using a threshold of a score of ≥1 (versus a score of 0), the sensitivity of the LqSOFA for predicting hospital admission was 30.6% (95% CI = 21.8% to 41.0%). The specificity was 84.7% (95% CI = 83.7% to 85.6%), the PPV was 3.0% (95% CI = 2.1% to 4.4%), and the NPV was 98.7% (95% CI = 98.4% to 99.0%). Using a threshold of ≥2 increased the specificity to 99.5% (95% CI = 99.3% to 99.7%) but reduced the sensitivity to 7.1% (95% CI = 3.3% to 14.6%).

**Table 2.** LqSOFA scoring system for all and febrile children for primary and secondary outcomes (full table can be viewed in Supplementary Table S7)

**Outcome, analysis, and threshold**	**Admitted, *n* (%)**	**Not admitted, *n* (%)**	**Sensitivity, % (95% CI)**	**Specificity, % (95% CI)**	**PPV, % (95% CI)**	**NPV, % (95% CI)**	**AUC (95% CI)**
**Primary outcome: hospital admission**							
Main (all children, *n* = 5568)							0.58 (0.53 to 0.63)
Score ≥1 (*n* = 867)	26 (3.0)	841 (97.0)	30.6 (21.8 to 41.0)	84.7 (83.7 to 85.6)	3.0 (2.1 to 4.4)	98.7 (98.4 to 99.0)	
Score ≥2 (*n* = 31)	6 (19.4)	25 (80.6)	7.1 (3.3 to 14.6)	99.5 (99.3 to 99.7)	19.4 (9.2 to 36.3)	98.6 (98.2 to 98.9)	
Sensitivity (febrile, *n* = 4221)							0.57 (0.52 to 0.62)
Score ≥1 (*n* = 690)	23 (3.3)	667 (96.7)	29.1 (20.3 to 39.9)	83.9 (82.7 to 85.0)	3.3 (2.2 to 5.0)	98.4 (97.9 to 98.8)	
Score ≥2 (*n* = 25)	5 (20.0)	20 (80.0)	6.3 (2.7 to 14.0)	99.5 (99.3 to 99.7)	20.0 (8.9 to 39.1)	98.2 (97.8 to 98.6)	

**Secondary outcome:^a^serious illness episode^b,c^**							
Main (all children, *n* = 5568)							0.55 (0.48 to 0.62)
Score ≥1	—	—	23 (14 to 39)	85 (84 to 85)	1 (1 to 1)	99 (99 to 99)	
Score ≥2	—	—	10 (4 to 22)	99 (99 to 99)	13 (5 to 29)	99 (99 to 99)	
Sensitivity (febrile, *n* = 4221)							0.54 (0.48 to 0.61)
Score ≥1	—	—	23 (14 to 39)	84 (83 to 85)	2 (1 to 3)	99 (99 to 99)	
Score ≥2	—	—	10 (4 to 22)	99 (99 to 99)	16 (6 to 35)	99 (99 to 99)	

a *For the secondary outcome, all percentages for predictive values are rounded to mask derivation of raw numbers, which need to be suppressed because of small numbers.*

b *Serious illness episode is defined as either a serious illness diagnosed in hospital within 2 days of GP consultation or a hospital admission lasting ≥1 night within 2 days of GP consultation^.^*

c *Admissions data could not be displayed because of unmasking of small numbers. AUC = area under receiver operating characteristic curve. LqSOFA = Liverpool quick Sequential Organ Failure Assessment. NPV = negative predictive value. PPV = positive predictive value.*

When the population was limited to febrile children, 79 (1.9%) were admitted to hospital (Table 1). The LqSOFA performed less well, with a sensitivity of 29.1% (95% CI = 20.3% to 39.9%) and specificity of 83.9% (95% CI = 82.7% to 85.0%) for a threshold of >1 (Table 2).

### Secondary outcome using LqSOFA

There were 42 (0.8%) children who had a serious illness episode (Table 1). For this outcome, a score of ≥1 had a sensitivity of 23% (95% CI = 14% to 39%), slightly worse than for the primary outcome, and a comparable specificity of 85% (95% CI = 84% to 85%). Sensitivity and specificity values were similar when the population was limited to febrile children (Table 2).

### National PEWS

**Figure 2.**
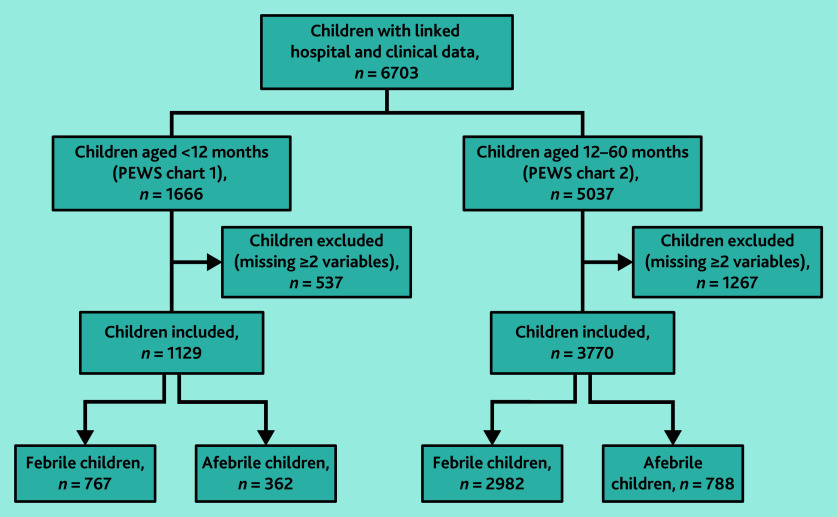
Sample cohorts for National PEWS analysis. PEWS = Paediatric Early Warning System.

Overall, 4899/6703 (73.1%) children were included in the analysis (Figure 2). There was a higher proportion of children aged <12 months missing data for ≥2 variables (32.2%, *n* = 537/1666) compared with children aged ≥12 months (25.2%, *n* = 1267/5037) (Supplementary Table S5). The children excluded from the analysis were younger and less likely to be febrile, with no significant difference in hospital admissions (Supplementary Table S6). The median age of included children was 7.2 months for children aged <12 months (PEWS chart 1) and 2.9 years for those aged ≥12 months (PEWS chart 2) (Table 1).

The most common PEWS score total was 1, accounting for 38.2% (*n* = 1872/4899) of score totals (Figure 1). A raised heart rate and raised respiratory rate were the most common reasons for scoring (data not shown).

### Primary outcome using National PEWS

A total of 79/4899 (1.6%) children were admitted to hospital, and the AUC was 0.66 (95% CI = 0.59 to 0.72) (Table 3 and Supplementary Table S8). Using a threshold of a score of ≥1 (versus a score of 0), the sensitivity was 81.0% (95% CI = 71.0% to 88.1%). The specificity was 32.5% (95% CI = 31.2% to 33.8%), the PPV was 1.9% (95% CI = 1.5% to 2.5%), and the NPV was 99.1% (95% CI = 98.4% to 99.4%). Using a threshold of ≥2 increased the specificity to 70.9% (95% CI = 69.6% to 72.2%) but reduced the sensitivity to 54.4% (95% CI = 43.5% to 65.0%).

**Table 3.** Combined National PEWS scoring system for all children aged <5 years and febrile children aged <5 years for primary and secondary outcomes (full table can be reviewed in Supplementary Table S8)

**Outcome, analysis, and threshold**	**Admitted, *n* (%)**	**Not admitted, *n* (%)**	**Sensitivity, % (95% CI)**	**Specificity, % (95% CI)**	**PPV, % (95% CI)**	**NPV, % (95% CI)**	**AUC (95% CI)**
**Primary outcome: hospital admission**							
Main (all children, *n* = 4899)							0.66 (0.59 to 0.72)
Score ≥1 (*n* = 3317)	64 (1.9)	3253 (98.1)	81.0 (71.0 to 88.1)	32.5 (31.2 to 33.8)	1.9 (1.5 to 2.5)	99.1 (98.4 to 99.4)	
Score ≥2 (*n* = 1445)	43 (3.0)	1402 (97.0)	54.4 (43.5 to 65.0)	70.9 (69.6 to 72.2)	3.0 (2.2 to 4.0)	99.0 (98.6 to 99.3)	
Score ≥3 (*n* = 685)	31 (4.5)	654 (95.5)	39.2 (29.2 to 50.3)	86.4 (85.4 to 87.4)	4.5 (3.2 to 6.4)	98.9 (98.5 to 99.1)	
Sensitivity (febrile, *n* = 3749)							0.64 (0.57 to 0.71)
Score ≥1 (*n* = 2581)	59 (2.3)	2522 (97.7)	79.7 (69.2 to 87.3)	31.4 (29.9 to 32.9)	2.3 (1.8 to 2.9)	98.7 (97.9 to 99.2)	
Score ≥2 (*n* = 1156)	39 (3.4)	1117 (96.6)	52.7 (41.5 to 63.7)	69.6 (68.1 to 71.1)	3.4 (2.5 to 4.6)	98.7 (98.1 to 99.0)	
Score ≥3 (*n* = 563)	29 (5.2)	534 (94.9)	39.2 (28.9 to 50.6)	85.5 (84.3 to 86.6)	5.2 (3.6 to 7.3)	98.6 (98.1 to 98.9)	

**Secondary outcome:^a^serious illness episode^b,c^**							
Main (all children, *n* = 4899)							0.60 (0.50 to 0.70)
Score ≥1	—	—	73.7 (58.0 to 85.0)	32.3 (31.0 to 33.7)	0.8 (0.6 to 1.2)	99.4 (98.8 to 99.7)	
Score ≥2	—	—	44.7 (30.1 to 60.3)	70.6 (69.3 to 71.9)	1.2 (0.7 to 1.9)	99.4 (99.0 to 99.6)	
Score ≥3	—	—	34 (21 to 50)	86 (85 to 70)	2 (1 to 3)	99 (99 to 100)	
Sensitivity (febrile, *n* = 3749)							0.59 (0.49 to 0.69)
Score ≥1	—	—	73.7 (58.0 to 85.0)	31.2 (29.7 to 32.7)	1.1 (0.8 to 1.6)	99.1 (98.4 to 99.5)	
Score ≥2	—	—	44.7 (30.1 to 60.3)	69.3 (67.8 to 70.8)	1.5 (0.9 to 2.3)	99.2 (98.7 to 99.4)	
Score ≥3	—	—	34 (21 to 50)	85 (84 to 86)	2 (1 to 4)	99 (99 to 100)	

a *For the secondary outcome, some percentages for predictive values are rounded to mask derivation of raw numbers that need to be suppressed owing to small numbers.*

b *Serious illness episode is defined as either a serious illness diagnosed in hospital within 2 days of GP consultation or a hospital admission lasting ≥1 night within 2 days of GP consultation.*

c *Admissions data could not be displayed because of unmasking of small numbers. AUC = area under receiver operating characteristic curve. NPV = negative predictive value. PEWS = Paediatric Early Warning System. PPV = positive predictive value.*

When the population was limited to febrile children, 74/4899 (1.5%) were admitted to hospital. The National PEWS performed slightly less well, with a sensitivity of 79.7% (95% CI = 69.2% to 87.3%) and specificity of 31.4% (95% CI = 29.9% to 32.9%) for a threshold score of ≥1 (Table 3 and Supplementary Table S8).

### Secondary outcome using National PEWS

There were 38 (0.8%) children who had a serious illness episode. For this outcome, a National PEWS score of ≥1 had a sensitivity of 73.7% (95% CI = 58.0% to 85.0%), slightly worse than for the primary outcome, and a specificity of 32.3% (95% CI = 31.0% to 33.7%). Sensitivity and specificity values were similar when the population was limited to febrile children (Table 3 and Supplementary Table S8).

## Discussion

### Summary

Overall, the results demonstrate that neither the LqSOFA nor the National PEWS are accurate for identifying children who are acutely unwell admitted to hospital within 2 days of a general practice consultation. Both scores demonstrated poor discrimination for predicting hospital admissions with an AUC range of 0.57 to 0.66. Neither tool performed well for identifying serious illnesses, with the CIs overlapping such that the scoring systems were no better than chance.

The LqSOFA score had a high specificity for a score of ≥2 (99.5%). This strong ‘rule in’ ability would highlight to GPs that children scoring ≥2 require urgent referral to hospital and should not be sent home. Conversely, the sensitivity is low; most children who were admitted would be missed using this threshold. An LqSOFA score of ≥1 had a slightly improved sensitivity of 30.6% but a poorer specificity of 84.7%.

For a scoring system to be useful to GPs it needs to have a high sensitivity, identifying all children who need admission so that those not flagged up can be confidently managed at home. The National PEWS had a better sensitivity than LqSOFA with a sensitivity of 81.0%, using a threshold of ≥1 point to ‘flag’ which children may require hospital admission. Although capturing the majority of children who were seriously ill, this would still miss 19.0% of children requiring admission. Specificity was low at 32.5%, meaning 67.5% of all presenting ill children would be flagged as needing admission.

### Strengths and limitations

This study is, to the authors’ knowledge, the first to evaluate the performance of an adjusted National PEWS score using general practice data, providing important results regarding the accuracy of this tool if it were to be introduced into general practice. A large dataset of children who were acutely unwell presenting to general practice including detailed presenting symptoms and signs was utilised.

In this study it was not possible to match all variables from either scoring system. There were differences in the categorisation of CRT that may have resulted in a greater number of children scoring for this variable. In addition, in this study data were not available for the PEWS variables blood pressure and oxygen requirement; however, these measurements are not routinely performed in general practice and would be of little use for GPs if included in this scoring system. A total of 26.9% of children had to be excluded because of missing variables. This could have created a selection bias as excluded children were younger; however, there was no difference in hospital admission rates.

Hospital admissions among children can be influenced by a variety of contextual factors and do not always indicate illness severity. Data were not available to allow exploration of biomedical or social reasons for admissions in the current study.

Additionally, the primary outcome of ‘admission within 2 days’ may have reduced the accuracy of these scoring systems; a shorter timeframe of 24 h is often used in hospital-based predictive studies to identify early deterioration. Furthermore, the National PEWS displays trends in scores that can be used to track deterioration. Including these factors in the current study could have improved the score’s sensitivity. However, the primary outcome reflected the intervals used in the pre-existing LqSOFA and National PEWS studies.

Finally, the current dataset may not have included children who were extremely unwell, requiring immediate transfer to hospital from the community. Nevertheless, decision tools would not be needed for these occasions as it would be clear to the clinician that urgent admission was required.

### Comparison with existing literature

The authors of the current study could not find other studies evaluating either of these scoring systems in a general practice setting. The LqSOFA derivation and validation cohorts included children attending a UK paediatric emergency department with a fever.^8^ Their primary outcome was an admission to critical care within 48 h. Using an LqSOFA score ≥1, they reported a sensitivity of 71.9% and specificity of 85.0% (AUC 0.81), demonstrating that the LqSOFA performs better in emergency departments than in general practice. This is likely owing to the difference in the stages of presentation and prevalence of serious illness between the two settings.

The current National PEWS has only recently been rolled out in hospitals nationwide. There is one previous study that assessed an earlier version alongside six other regional PEWS within an emergency department.^13^ For the primary outcome of ‘critical care admission within 48 h’ the National PEWS performed well, with sensitivities and specificities of 89.6% and 84.7%, respectively, using a threshold of ≥5. No data were presented for lower score thresholds.

One scoring system developed and validated in primary care in Belgium was identified. This ‘four-step decision tree’ had a sensitivity of 100% and specificity of 83.6% when it was validated. However, the validation study included children up to age 16 years and from emergency department and outpatient settings.^15^^,^^16^ This warrants further exploration in a UK cohort of younger children presenting to general practice.

### Implications for research and practice

The current NICE-recommended clinical tool used in general practice is the ‘traffic light’ system, which has poor sensitivity (58.8%) and specificity (68.5%) for identifying children who are seriously ill.^7^ The two scores evaluated here are simpler to use, with objective variables in comparison with the many subjective variables in the traffic light system. However, neither performed well.

If the National PEWS had performed well in general practice this could have provided a common language across prehospital and hospital settings, improved continuity of care, and potentially improved outcomes in children. However, the current study has shown that an adapted version of the National PEWS performs poorly for predicting admissions from general practice within 2 days and should not be incorporated nationally into general practices for the assessment of children who are acutely unwell as it stands. It is possible that the full PEWS may have performed better than the current authors’ adapted version; however, blood pressure is not commonly measured in children in general practice nor is oxygen commonly administered.

Further research is needed to derive and validate an accurate scoring system in general practice that is both easy to use and accurate. This may involve validation of an existing score or the development of a new or adjusted early warning score using prospective general practice data and including qualitative work with primary and secondary care clinicians.

## Data

This study makes use of anonymised data held in the Secure Anonymised Information Linkage system, which is part of the national e-health records research infrastructure for Wales. NHS Digital data re-use statement: © (2019) the Health and Social Care Information Centre. Re-used with the permission of the Health and Social Care Information Centre. All rights reserved. The patient datasets analysed in this study cannot be made available because of confidentiality and data-sharing agreements in place. This work uses data provided by patients and collected by the NHS as part of their care and support. #datasaveslives
